# 575. Safety and Immunogenicity of mRNA-1403, a Multivalent Norovirus mRNA Vaccine, in Healthy Adults: Interim Results of a Phase 1/2, Randomized, Observer-Blind, Placebo-Controlled, Dose-Ranging Trial

**DOI:** 10.1093/ofid/ofae631.013

**Published:** 2025-01-29

**Authors:** Till Schoofs, Brooke A Bollman, Kevin Mancini, Wenlin Yuan, Lauren Bailey, Tin Bartholomew, Esther Levine, Katherine B Carlson, Alexander Rumyantsev, Meklit Workneh, Doran Fink, Jaap Oostendorp

**Affiliations:** Moderna, Inc, Cambridge, MA; Moderna, Inc, Cambridge, MA; Moderna, Inc, Cambridge, MA; Moderna, Inc, Cambridge, MA; Moderna, Inc, Cambridge, MA; Moderna, Inc, Cambridge, MA; Moderna, Inc, Cambridge, MA; Moderna, Cambridge, Massachusetts; Moderna, Inc, Cambridge, MA; Moderna, Inc, Cambridge, MA; Moderna, Inc, Cambridge, MA; Moderna, Inc, Cambridge, MA

## Abstract

**Background:**

Norovirus (NoV) is a leading cause of acute gastroenteritis worldwide and is associated with significant disease burden across all ages. Currently, there is no licensed NoV vaccine. NoV’s high genetic diversity coupled with genotype-specific immune responses indicate that a multivalent approach may be required to generate a broadly protective vaccine. A trivalent mRNA-based NoV vaccine candidate (mRNA-1403), consisting of mRNAs encoding for the major capsid protein (VP1) of 3 globally prevalent NoV genotypes (GII.4, GI.3 and GII.3) formulated in a lipid nanoparticle, is in development.

**Methods:**

In this ongoing Phase 1/2, randomized, placebo-controlled, observer-blind, dose-ranging study, safety and immunogenicity of mRNA-1403 are being evaluated in healthy adults 18 to 80 years of age (NCT05992935). In Phase 1, healthy younger (18-49 years) and older (60-80 years) adults were randomized to receive 1 of 4 dose levels of mRNA-1403 given as 2 doses, 1 dose level of mRNA-1403 given as 1 dose, or Placebo. In Phase 2, 3 dose levels of mRNA-1403 given as a single-dose schedule were selected for further evaluation in a larger sample size of healthy adults 18-80 years of age.

**Results:**

In Phase 1, 335 healthy adults received 1 or 2 doses of mRNA-1403 or Placebo. mRNA-1403 was well-tolerated across all dose levels with no safety concerns identified through 8 months of follow-up from dose 1. A single injection of mRNA-1403 elicited robust levels of serum histo-blood group antigen (HBGA)-blocking antibodies and binding antibodies against vaccine-matched NoV genotypes at 1 month post-dose across all dose levels tested. In Phase 2, 616 healthy adults received a single injection of 1 of 3 selected dose levels of mRNA-1403 or Placebo. Data in this larger sample size of healthy younger and older adults through 1 month post-dose confirmed that a single-dose schedule of all 3 dose levels of mRNA-1403 was well-tolerated, safe and immunogenic.

**Conclusion:**

Available data from this ongoing Phase 1/2 study show that mRNA-1403 was generally safe and well-tolerated, and induced antigen-specific immune responses at all dose levels in both younger and older healthy adults. These results informed dose selection for a Phase 3 efficacy study in adults.

**Disclosures:**

**Till Schoofs, MD**, Moderna, Inc: Employee|Moderna, Inc: Stocks/Bonds (Public Company) **Brooke A. Bollman, PhD**, Moderna, Inc: Employee|Moderna, Inc: Stocks/Bonds (Public Company) **Kevin Mancini, MS**, Moderna, Inc: Employee|Moderna, Inc: Stocks/Bonds (Public Company) **Wenlin Yuan, MS, PhD**, Moderna, Inc: Employee|Moderna, Inc: Stocks/Bonds (Public Company) **Lauren Bailey, PhD**, Moderna, Inc: Employee|Moderna, Inc: Stocks/Bonds (Public Company) **Tin Bartholomew, MA**, Moderna, Inc: Employee|Moderna, Inc: Stocks/Bonds (Public Company) **Esther Levine, MD**, Moderna, Inc: Employee|Moderna, Inc: Stocks/Bonds (Public Company) **Katherine B. Carlson, PhD, MPH**, Moderna, Inc: Employee|Moderna, Inc: Stocks/Bonds (Public Company) **Alexander Rumyantsev, MD, PhD**, Moderna, Inc: Employee|Moderna, Inc: Stocks/Bonds (Public Company) **Meklit Workneh, MD, MPH**, Moderna, Inc: Employee|Moderna, Inc: Stocks/Bonds (Public Company) **Doran Fink, MD, PhD**, Moderna, Inc: Employee|Moderna, Inc: Stocks/Bonds (Public Company) **Jaap Oostendorp, PhD**, Moderna, Inc: Employee|Moderna, Inc: Stocks/Bonds (Public Company)

